# Fitness Trade-Offs in Competence Differentiation of *Bacillus subtilis*

**DOI:** 10.3389/fmicb.2016.00888

**Published:** 2016-06-07

**Authors:** Melih Yüksel, Jeffrey J. Power, Jan Ribbe, Thorsten Volkmann, Berenike Maier

**Affiliations:** Department of Physics, University of CologneKöln, Germany

**Keywords:** competence, phenotypic heterogeneity, fitness, antibiotic tolerance, persistence

## Abstract

In the stationary phase, *Bacillus subtilis* differentiates stochastically and transiently into the state of competence for transformation (K-state). The latter is associated with growth arrest, and it is unclear how the ability to develop competence is stably maintained, despite its cost. To quantify the effect differentiation has on the competitive fitness of *B. subtilis*, we characterized the competition dynamics between strains with different probabilities of entering the K-state. The relative fitness decreased with increasing differentiation probability both during the stationary phase and during outgrowth. When exposed to antibiotics inhibiting cell wall synthesis, transcription, and translation, cells that differentiated into the K-state showed a selective advantage compared to differentiation-deficient bacteria; this benefit did not require transformation. Although beneficial, the K-state was not induced by sub-MIC concentrations of antibiotics. Increasing the differentiation probability beyond the wt level did not significantly affect the competition dynamics with transient antibiotic exposure. We conclude that the competition dynamics are very sensitive to the fraction of competent cells under benign conditions but less sensitive during antibiotic exposure, supporting the picture of stochastic differentiation as a fitness trade-off.

## Introduction

Transformation is the process of uptake and inheritable integration of DNA from the environment. The evolutionary benefit of transformation is still a matter of debate (Redfield, 2001; Chen and Dubnau, 2004). Three major hypotheses for the benefit of transformation (and recombination in general) are reducing maladaptation due to the accumulation of deleterious mutations, reducing clonal interference, and acquisition of adaptive alleles from other species (Fisher, 1930; de Visser and Elena, 2007). On the other hand, competence for transformation is likely to induce costs including the physiological cost of generating machines for DNA exchange, and the genetic cost of acquiring deleterious alleles and reduction of fitness in the presence of strong epistasis (Moradigaravand and Engelstädter, 2013; Nowak et al., 2014). While some species are continuously competent for transformation, others differentiate into a distinct physiological state, the state of competence for transformation (Johnston et al., 2014). *Streptococcus pneumoniae* and *Bacillus subtilis* transiently switch into the state of competence during certain growth phases.

Interestingly, competence in *S. pneumoniae* can be induced by the application of antibiotics (Prudhomme et al., 2006; Slager et al., 2014). This observation provoked the question, whether the state of competence confers a benefit in the presence of antibiotics that is independent of transformation (Claverys et al., 2006; Engelmoer and Rozen, 2011). Indeed, it was shown that under treatment with the translation inhibitor streptomycin, competent cells were fitter and this selective advantage was independent of transformation (Engelmoer and Rozen, 2011). Importantly, competence does not confer growth arrest in *S. pneumoniae* (Engelmoer and Rozen, 2011). The situation is very different for *B. subtilis*. Competent *B. subtilis* cells are growth-arrested (Haijema et al., 2001; Briley et al., 2011). When stationary cells are diluted into fresh medium, non-competent cells rapidly resume growth whereas competent cells experience a lag-phase lasting for about 2 h (Briley et al., 2011). It is currently unclear, however, whether growth arrest confers a large cost under natural conditions considering that competence is a stationary state phenomenon. Mechanistically, growth arrest is caused by a failure to assemble replisomes (Briley et al., 2011; Hahn et al., 2015). The late competence protein ComGA has been shown to be involved in growth arrest and inhibits rRNA synthesis (Haijema et al., 2001; Hahn et al., 2015). For *B. subtilis*, the state of competence confers a benefit under penicillin treatment (Nester and Stocker, 1963). Johnsen et al. proposed that competence may be selected for because growth arrest conferred persistence under episodic treatment with penicillin (Johnsen et al., 2009). Computer simulations predicted a benefit, and this benefit was verified experimentally using time-kill kinetics and head-to-head competition experiments between a laboratory strain of *B. subtilis* with a fraction of ~15% competent cells and a non-competent strain (Johnsen et al., 2009). Recent time-kill experiments with oxolinic acid and kanamycin revealed increased antibiotic tolerance of wt cells as compared to non-competent *B. subtilis* (Hahn et al., 2015).

There is an interesting hypothesis proposing that *B. subtilis* may minimize the cost of competence by restricting competence to a subpopulation. During the stationary growth phase, *B. subtilis* switches into the state of competence (aka K-state) with a strongly increased probability compared to the exponential growth phase (Dubnau and Losick, 2006; Veening et al., 2008). In particular, cells transiently differentiate into the state of competence for transformation (Süel et al., 2006). As a consequence of transient differentiation, only a fraction of cells are competent at a given point in time, generating phenotypic heterogeneity. The molecular mechanism underlying phenotypic heterogeneity is well understood. Development of competence is governed by the master regulator ComK (Hahn et al., 1994) and induced by quorum sensing and nutrient limitation (Solomon et al., 1996). Bimodal behavior is generated by a cooperative autocatalytic feedback loop of the *comK* gene (Maamar and Dubnau, 2005; Smits et al., 2005). Each cell generates a basal level of ComK, and this level increases slightly during the entry into the stationary growth phase (Leisner et al., 2007). On average, each cell maintains one *comK* mRNA molecule (Maamar et al., 2007). This low copy number generates high noise, and the amplitude of this noise has been shown to correlate with the probability of differentiation into the state of competence (Maamar et al., 2007; Süel et al., 2007). The basal level of ComK is influenced by the repressor of *comK, rok* (Leisner et al., 2008; Maier, 2012). Deletion of *rok* has a very strong impact on the probability of differentiation (Hoa et al., 2002). Nearly all cells lacking this repressor develop competence (Leisner et al., 2009). It has been demonstrated that the probability of differentiating into and escaping from the state of competence can be controlled by fine-tuning the expression levels of *comK* and *comS* (Espinar et al., 2013). ComS increases the probability for competence development by inhibiting the proteolysis of ComK (Turgay et al., 1998). When basal ComK levels exceed a threshold concentration, ComK levels rise in a switch-like fashion and the cell switches into the K-state (Maamar and Dubnau, 2005; Smits et al., 2005). The duration of competence is highly variable (Cagatay et al., 2009) in agreement with noise generated by the low copy number of ComS molecules (Mugler et al., 2016).

Natural isolates of *B. subtilis* most likely show a variety of differentiation probabilities into the K-state. For example, plasmids affect the probability of competence development. The non-conjugative plasmid pBS32 found in the undomesticated strain NCIB3610 interferes with competence development via ComI (Konkol et al., 2013) and the conjugative plasmid pLS20 encodes of the repressor of *comK, rok*_*LS*20_ (Singh et al., 2012). It has been proposed that phenotypic heterogeneity is likely to act as a bet-hedging strategy (Veening et al., 2008; Wylie et al., 2010; Hahn et al., 2015) which enables the major fraction of the population to grow fast under benign conditions. Under conditions of stress, however, the small subpopulation of growth-arrested K-state cells may be at an advantage and ensure the survival of the population. Direct proof has not been provided to our knowledge.

Here, we scrutinized fitness trade-offs of the K-state. In particular, we tested the hypothesis that stochastic differentiation into the K-state minimizes fitness costs while increasing the rate of survival in the face of stress. To this end, we characterized the competition dynamics of *B. subtilis* strains that entered the K-state with different probabilities under benign conditions and in the presence of bactericidal and bacteriostatic antibiotics. Under benign conditions the fitness cost increased as a function of the probability of differentiation. In the presence of antibiotics, the K-state was beneficial, but the fraction of K-state cells had little effect on the competition dynamics. Our data indicates that a small fraction of K-state cells are enough to ensure re-growth after antibiotic exposure.

## Materials and methods

### Bacterial strains and growth conditions

All strains used in this study were derived from strain BD630 and are listed in Table 1. *B. subtilis* strains were grown either in liquid LB medium or in competence medium (Albano et al., 1987), supplemented with 0.5% glucose, 50 μg/ml L-histidine, L-leucine, L-methionine, and chloramphenicol (5 μg/ml), kanamycin (5 μg/ml), or spectinomycin (100 μg/ml) at 37°C. Growth was monitored either by measuring the OD_600_ on a Genesys 10S UV-VIS spectrophotometer (Thermo Scientific) or by using an Infinite M200 plate reader (Tecan, Männedorf, Switzerland). Competent cells were prepared as described previously and transformed by following the standard protocol (Albano et al., 1987). Bs139 (wt *gfp*) was generated by transforming BD630 with genomic DNA of AR16 (obtained from the *B. subtilis* Stock Center; Rosenberg et al., 2012).

**Table 1:** **Strains used in this study**.

**Strain**	**Relevant genotype**	**Source/Reference**
BD630	*his leu met*	–
BD2711	*his leu met, P_*comK*_-gfp (CBL^*b*^, cat^*a*^)*	Haijema et al., 2001
Bs056	*his leu met, rok- (kan^*a*^)*	This study
Bs075	*his leu met, comK- (kan^*a*^)*	This study
Bs139	*his leu met, amyE::P_*rrnE*_-gfpmut2-spc^*a*^*	This study
Bs140	*his leu met, amyE::P_*rrnE*_-gfpmut2-spc^*a*^, comK- (kan^*a*^)*	This study
Bs141	*his leu met, amyE::P_*rrnE*_-gfpmut2-spc^*a*^, rok- (kan^*a*^)*	This study
Bs142	*his leu met, rok- (kan^*a*^), comK- (spc^*a*^)*	This study
Bs144	*his leu met, rok- (kan^*a*^), comEC- (cat^*a*^)*	This study
BD3836	*his leu met, amyE::P_*hs*_ comK (spc^*a*^)*	Maamar and Dubnau, 2005

a *kan, cat, and spc describe resistance to kanamycin, chloramphenicol, and spectinomycin, respectively*.

b *Inserted by Campell like integration*.

The *rok* deletion mutant (Bs056) was generated as follows. The 400 bp region upstream of the *rok* gene was amplified from BD630 chromosomal DNA introducing a SacI restriction site, and the 700 bp region downstream of the *rok* gene was amplified introducing an EcoRI restriction site. The aminoglycoside phosphotransferase type III gene (*nptIII*), encoding for an aminoglycoside phosphotransferase, was fused at the 5′-end with the upstream region and at the 3′-end with the downstream region. The PCR fusion was inserted between the SacI and EcoRI sites of pBluescriptIIKS resulting in *pRok::kan*. *pRok::kan* was transformed into BD630 to generate Bs056 (Δ*rok*).

Bs144 (Δ*rok*Δ*comEC*) was generated as follows. The 460 bp region upstream of the *comEC* gene was amplified from BD630 chromosomal DNA introducing a SacI restriction site, and the 460 bp region downstream was amplified introducing an EcoRI restriction site. The chloramphenicol acetyltransferase gene (*cat*), encoding for a chloramphenicol acetyltransferase, was fused at the 5′-end with the upstream region and at the 3′-end with the downstream region. The PCR fusion was inserted between the SacI and EcoRI sites of pBluescriptIIKS resulting in *pComEC::kan*. *pComEC::kan* was transformed into Bs056 to generate Bs144 (*rok*Δ*comEC*).

Bs075 (Δ*comK*) was generated as follows. The 900 bp regions upstream and downstream of *comK* were amplified from BD630 chromosomal DNA. The *nptIII* gene was fused at the 5′-end with the upstream region and at the 3′-end with the downstream region. This PCR fusion was transformed into BD630 to generate Bs075 (Δ*comK*).

Bs142 (Δ*rok*Δ*comK*) was generated as follows. The 900 bp regions upstream and downstream of *comK* were amplified by using BD630 chromosomal DNA. The spectinomycin adenyltransferase gene (*aadA*), encoding for a spectinomycin adenyltransferase, was fused at the 5′-end with the upstream region and at the 3′-end with the downstream region. This PCR fusion was transformed into Bs056 to generate Bs142 (Δ*rok*Δ*comK*).

### Competition assay during outgrowth

Overnight cultures of both competitors were diluted to OD_600_ 0.1 in fresh competence medium and were grown separately to the stationary growth phase T_2_ (i.e., 2 h after the entry into the stationary growth phase). Cells were diluted 10-fold into fresh pre-warmed competence medium containing different concentrations of ampicillin, erythromycin, and rifampicin, respectively, and mixed in a 1:1 ratio. Various concentrations of each antibiotic were used, depending on the particular experiment; see figures for details. After 1 h incubation at 37°C with agitation, antibiotics were washed out by centrifuging and re-suspending twice. Cells were incubated for 24 h. Cell suspensions of 10 μl were taken at several time points and mixed with 1 ml PBS. In all competition experiments, one of the strains was labeled fluorescently with *amyE::P*_*rrnE*_*-gfpmut2*. The fraction of the fluorescent reporter strain was measured using a BD Canto II flow cytometer (BD Bioscience, Franklin Lakes, USA) equipped with three solid–state lasers at 405, 488, and 561 nm. For detection of GFP fluorescence a 530/30 filter was used. The photomultiplier voltage for forward scatter (FSC), side scatter (SSC), and GFP was set at 100, 351, and 450, respectively. To exclude particles smaller than *B. subtilis*, the threshold for FSC was set at 250. At least 10,000 cells/sample were analyzed using BD FACSDiva 6 software (BD Bioscience, Franklin Lakes, USA).

During exponential growth, *B. subtilis* forms chains. FSC and SSC signals of chains observed in the flow cytometer were slightly increased compared to single cells. All events were counted as single cells because flow cytometry measures events and cannot distinguish between single cells and groups of cells forming a chain. Since only non-competent exponentially growing cells form chains, the ratio between cells with a higher probability of entering the K-state and those with a lower probability of entering the K-state, may be overestimated. This would indicate that the competitive fitness of K-state forming cells may be overestimated. However, this overestimation does not affect the major conclusions for the following reasons. Under benign conditions, overestimation of the fraction of K-state leads to an underestimation of the fitness cost, i.e., the fitness cost of competence may even be larger. With antibiotic treatment, the ratio between K-state cells and non K-state cells may be overestimated at early time points. However, the benefit of the K-state clearly persisted within the stationary phase where *B. subtilis* does not form chains. As a further control supporting the benefit of the K-state during transient antibiotic exposure, we analyzed the re-initiation probability of growth at the single cell level, confirming that K-state cells are more likely to re-initiate growth after antibiotic exposure.

### Competition assay in the stationary phase

Overnight cultures of both competitors were diluted to OD_600_ 0.1 into fresh competence medium and were grown separately to stationary growth phase T_0_ (entry into the stationary phase). Cells were diluted 10-fold into conditioned medium. Conditioned medium was obtained by centrifuging (17,600 × g, 3 min) and filtering (0.2 μm pore sterile filters, Sarstedt) T_0_ medium. Conditioned media was always made fresh for each experiment. Cells were incubated for 24 h. The fraction of fluorescent cells was determined following the description for the outgrowth competition assay.

### Competition assay with IPTG-inducible *comK*

Overnight cultures of both competitors were diluted to OD_600_ 0.1 in fresh competence medium and were grown separately for 2.5 h and subsequently induced with IPTG (concentrations detailed in Figure S8) for 30 min. After IPTG was washed out by centrifuging and re-suspending twice, cells were diluted 10-fold into fresh pre-warmed competence medium containing different concentrations of ampicillin and mixed in a 1:1 ratio. After 1 h incubation at 37°C with agitation, antibiotics were washed out by centrifuging and re-suspending twice. Cells were incubated for 4 h. Cell suspensions of 10 μl were taken at several time points and mixed with 1 ml PBS for quantification of the ratios using flow cytometry.

### Time lapse microscopy

For re-initiation of growth, an overnight culture of BD2711 was diluted to OD_600_ 0.1 in fresh competence medium and grown to T_2_, where the number of competent cells reaches its maximum (Leisner et al., 2007). Cells were diluted 10-fold into fresh pre-warmed competence medium and sandwiched between a glass cover slide and a 1.5 mm-thick gel made of phosphate buffered solution (PBS, Sigma) with 1% Bacto agar (BD). The glass cover slide was sealed onto the flow chamber using picodent twinsil (Picodent) confining the PBS gel between the cover slide and a polydimethylsiloxane (PDMS) pad (Ducret et al., 2009; Boulineau et al., 2013; Figure S1).

For stationary phase growth, the overnight culture of BD2711 was diluted to OD_600_ 0.1 in fresh competence medium and grown to T_0_. Cells were diluted 20-fold in conditioned medium. The diluted sample was sandwiched between a glass cover slide and a 1.5 mm-thick polyacrylamide gel. The polyacrylamide gel was made using 20% acrylamide/bis-acrylamide (29:1 ratio, Sigma), 0.1% ammonium persulfate (Roth), and 0.1% TEMED (Roth). After waiting several hours to ensure the gel had completely solidified, spacers were removed and the gel (with microscope slides) was soaked in double-distilled water (ddH_2_O) for several minutes. This short soaking phase prevented the gel from tearing when the microscope slides were removed. The gel was cut to the appropriate dimensions and soaked twice in ddH_2_O for at least 5 h. The cut gels were stored in fresh ddH_2_O until use (Nghe et al., 2013). Similar to the re-initiation of growth experiment, the glass cover slide with polyacrylamide gel and diluted sample was sealed onto the flow chamber using picodent twinsil, confining the polyacrylamide gel between the cover slide and the structured PDMS pad.

The in-house flow chamber was mounted onto an inverted microscope (Nikon Eclipse TE2000-E). The flow chamber was made of polyoxymethylene with a 6 × 56 × 1 mm channel where a 6 × 26 mm hole was cut into the center. The hole was sealed using PDMS which formed a structured pad with an array of 0.5 μm tall pillars spaced 1 μm apart from each other. The channel was sealed with the glass cover slide and gel, as outlined above. Medium was flushed through the chamber at a rate of 10 μl/min during image acquisition and at speeds of up to 500 μl/min to initially fill the chamber or flush the chamber of antibiotics. For re-initiation of growth, a syringe pump was used to control chamber flow rates and fresh competence medium and ampicillin (0.5 mg/ml), erythromycin (8 μg/ml), or rifampicin (15 μg/ml) were used as medium. For stationary phase growth, the batch culture (unfiltered at 40°C—to obtain 37°C when the culture reached the chamber—and constantly stirred), which had been used to create the conditioned medium for the dilution, was used as medium with a peristaltic pump.

DIC images were taken at 10 min intervals for up to 24 h. For stationary phase growth, images were taken according to the “correlation images” method (Julou et al., 2013). A z-stack height of ±3 μm, vertical step of 250 nm, and standard deviation of 700 nm were used.

### Image analysis

First, correlation images were calculated for a set of z-stack images, resulting in a final image where the cells have a low grayscale-intensity and are surrounded by a high grayscale-intensity contour. A threshold was then applied to the correlation images using a script developed in conjunction with Anthony et al. (2008). After applying the threshold, images were imported into Schnitzcells (Young et al., 2012) for tracking, and lineages were analyzed using an in-house Matlab script. In short, genealogy trees were drawn for each image set and generation time for every dividing cell was calculated, taking note of when a cell had become competent prior to division. Competence was monitored using P_*comK*_*-gfp* (Bs140). A number of cells developed competence, but did not divide during the time frame of the experiment. These cells were not included in the calculated mean generation time for competent cells. Since such cells include those with particularly long generation times, the mean generation time of cells that run through a competence period is underestimated.

## Results

### K-state confers fitness cost during the stationary growth phase

**Figure 1:**
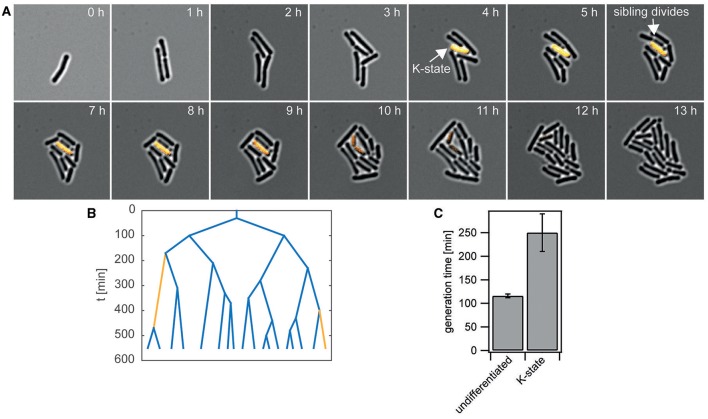
**Differentiation into the K-state is associated with growth arrest. (A)** Typical time lapse of *P*_*comK*_ *gfp* (BD2711) grown in conditioned stationary state medium, starting with T_0_ medium. Bright-field and fluorescence images are merged showing differentiated bacteria in orange. **(B)** Typical genealogy of cells after T_0_ grown in the flow chamber and supplied with conditioned medium. Blue, non K-state cells; orange, cells differentiating into the K-state. **(C)** Average generation times.

Competent *B. subtilis* are growth-arrested (Haijema et al., 2001), but cells almost exclusively differentiate into the K-state when they enter the stationary phase. Therefore, it was unclear whether growth arrest during the stationary phase conferred a fitness cost, since growth was absent under conditions favoring differentiation. To address this point, we set up flow chamber experiments that enabled us to monitor differentiation into the K-state under stationary-state conditions (see Materials and Methods). While cells were immobilized below an agar pad, conditioned medium from a shaking culture of *B. subtilis* wt was continuously flushed over the agar pad. Using the reporter strain *P*_*comK*_*gfp* (BD2711), we distinguished between competent and non-competent cells (Figure 1A). Under these conditions the generation time of non K-state cells was *t*_*nK*_ = *(116* ± *4*) min (Figures 1B,C). Even though net growth was observed in this experimental setup, bacteria transiently differentiated into the K-state (Figures 1A,B), as observed previously (Süel et al., 2006). Figure 1A shows an example where the K-state cell did not grow or divide while its sibling (from the previous division event) continued to grow and divide. The average generation time of cells that ran through a period of differentiation was considerably increased to *t*_*K*_ = *(250* ± *40*) min.

In the next step, we set out to determine how the probability of differentiating into the K-state affected the relative fitness in the stationary phase. To this end, we performed head-to-head competition experiments between strains with varying differentiation probability. The fraction of wt cells in early stationary phase is 15%. Differentiation is fully inhibited in a *comK* deletion strain, whereas nearly 100% of the *rok* deletion strain differentiates. To determine the selection coefficients, the competitor strains were individually grown to T_0_, then mixed in a 1:1 ratio. T_0_ denotes the entry into the stationary phase and is defined as the time point at which the OD_600_ switches from exponential increase to a nearly constant value. The mixed population was incubated for one day and during this time, the OD_600_ was monitored continuously. For all competition experiments shown, one of the competitor strains carried a *gfp* reporter. The reporter strain was used to quantify the fractions of both competitors using flow cytometry at various time points during the competition experiments (see Materials and Methods, competition assay during stationary phase).

**Figure 2:**
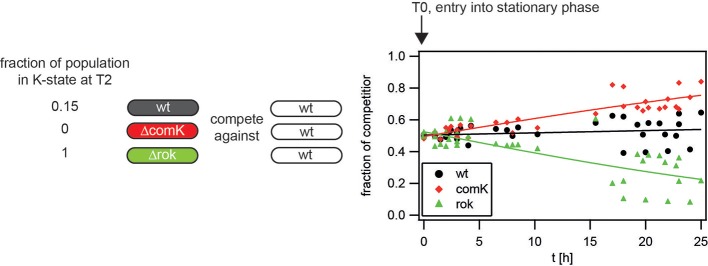
**Determination of selection coefficients in the stationary state**. Competitors were grown separately to T_0_, diluted into fresh competence medium, and mixed in a 1:1 ratio, as outlined in competition assay in the stationary phase. The fraction of wt (BD630, black circle), hyper-competent Δ*rok* (Bs056, green triangle), and non-competent Δ*comK* (Bs075, red diamond) competing against wt *gfp* (Bs139) cells are plotted. Full lines: best fit to replicator equation. The data were obtained from at least three independent experiments for each condition.

The entire competition experiment ran in the stationary growth phase. To quantify the cost, we assumed that the difference *s*_*ij*_ = *r*_*i*_ − *r*_*j*_, between the effective growth rates *r*_*i*_ and *r*_*j*_ of the competitors, was constant. Using the replicator equation, we determined the selection coefficients *s*_*ij*_ by fitting the data in Figure 2 with $x_{i}\left(t\right)\;=\;\lambda e^{st}∕\left(1\;+\;\lambda e^{st}\right)$, where *x*_*i*_(*t*) is the frequency of type *i* at time *t*, and λ is the ratio of initial frequencies of both competitors (Nowak, 2006).

As a control, wt (BD630) was competed with the reporter strain wt *gfp* (Bs139) and we found that the fractions remained close to 0.5 during the time course of the experiment. The selection coefficient was *s*_*wt*_ = *(0.006* ± *0.004)* h^−1^, i.e., close to 0 (Figure 2). When non-competent Δ*comK* (Bs075) competed with wt *gfp*, the fraction of wt cells decreased continuously with *s*_Δ*comK*_ = *(0.047* ± *0.005)* h^−1^. During competition between hyper-competent Δ*rok* (Bs056) and wt *gfp*, the wt dominated and the selection coefficient was *s*_Δ*rok*_ = *(*−*0.052* ± *0.007)* h^−1^.

These experiments show that even in the stationary phase where overall growth is strongly reduced, competence development is associated with a strong cost. This cost is tuned gradually as a function of the probability of differentiating into the K-state.

### The probability of differentiating into the K-state governs the competition dynamics during outgrowth

When stationary-state *B. subtilis* is diluted into fresh medium containing low levels of pheromones and high levels of nutrients, the K-state confers a fitness cost, because it extends the lag phase by ~2 h while non-competent cells rapidly resume growth (Briley et al., 2011). Here, we addressed the question whether the probability of differentiation into the K-state affected competitive fitness in a dosage-dependent way. We expect that the lag-phase and different growth rates during the entry and within the stationary phase govern the relative fitness. Therefore, the growth rates of the individual strains give little information about the relative fitness. Instead the competition dynamics between strains that have different differentiation probabilities was monitored using flow cytometry. Both competitors were grown separately to the stationary growth phase. The probability of being competent is maximal at T_2_(i.e., 2 h after the entry into stationary growth phase). Cells were harvested at T_2_, diluted into fresh competence medium, and subsequently the competitors were mixed in a 1:1 ratio. Competition experiments were run as explained in the Materials and Methods, competition assay during outgrowth.

**Figure 3:**
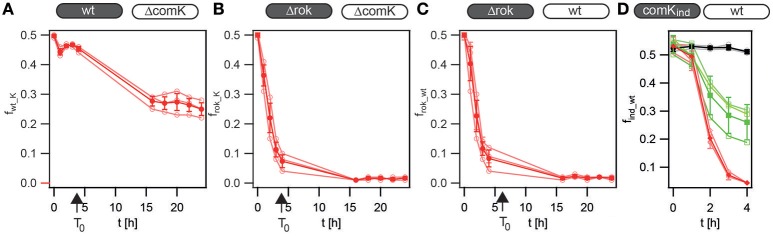
**The probability of differentiating into the K-state governs the cost of competence during outgrowth. (A–C)** Competitors were grown separately to T_2_, diluted into fresh competence medium, and mixed in a 1:1 ratio, as outlined in competition assay during outgrowth. **(A)** Fraction of wt (BD630) cells competed against non-competent Δ*comK gfp* (Bs140) cells. **(B)** Fraction of Δ*rok* (Bs056) cells competed against non-competent Δ*comK gfp* cells. **(C)** Fraction of Δ*rok* cells competed against wt *gfp* (Bs139) cells. T_0_: time point of entry into stationary state. **(D)** Competitors were grown separately for 2.5 h, induced, washed, and mixed in a 1:1 ratio. Fraction of *comK*_ind_ (BD3836) cells competing against wt *gfp (Bs139)* cells. Black: no induction, green: 50 μM IPTG, red: 200 μM IPTG. Open symbols, three independent experiments; closed symbols, average and standard deviation.

When wt cells (with an initial fraction of ~15% competent cells) competed with the non-competent Δ*comK gfp* (Bs140) cells, the fraction of wt cells dropped to *f*_*wt*_ = *(0.46* ± *0.01)* after 2 h of competition and remained roughly constant throughout the exponential phase (Figure 3A). After ~4 h, the population entered into the stationary phase. During the stationary phase, *f*_*wt*_ decreased further to *f*_*wt*_ = *(0.25* ± *0.02)* after 24 h since the remaining wt cells were able to become competent in the stationary phase.

When Δ*rok* cells (with an initial fraction of ~100% competent cells) competed with the non-competent Δ*comK gfp* cells, the fraction of Δ*rok* cells dropped continuously during the exponential phase to *f*_*rok_K*_ = *(0.07* ± *0.02)* after 4 h (Figure 3B). In the stationary phase, the fraction decreased further to *f*_*rok_K*_ = *(0.02* ± *0.01)* after 24 h.

The dynamics of the competition between Δ*rok* cells and wt *gfp* cells was reminiscent of the previous experiment with *f*_*rok_wt*_ = *(0.08* ± *0.03)* after 4 h and *f*_*rok_wt*_ = *(0.02* ± *0.01)* after 24 h (Figure 3C).

The probability of entering the K-state can also be controlled by putting *comK* under an inducible promoter. Competition experiments between a strain that had a second copy of *comK* under the IPTG-inducible hyper-SPANK promoter (BD3836) (Maamar and Dubnau, 2005) and the wild type were performed at various levels of induction (Figure 3D). The concentrations of IPTG were chosen to generate different fractions of K-state cells (Maamar and Dubnau, 2005). For this specific competition, cells were induced during the exponential growth phase, washed, mixed, and treated with antibiotics for 1 h. After removal of the antibiotics, the OD_600_ of the mixed culture and the fractions of the competitors were monitored for 3 h. In the absence of induction and antibiotics, the fractions of the competitors remained at *f*_*ind_wt*_ ≈ *0.5* (Figure 3D). With increasing concentrations of IPTG, which have been shown to affect the fraction of K-state cells (Maamar and Dubnau, 2005), the mutant fraction decreased more rapidly with increasing ComK levels.

Control experiments were performed to ensure that the GFP reporter did not affect the competition dynamics. For this, competition experiments between wt and wt *gfp* (Figure S2A) and between Δ*comK* and Δ*comK gfp* (Figure S2B), respectively, were performed. For both experiments, the fractions of the competitors remained close to 0.5, confirming that the reporter did not affect the competition dynamics.

In the K-state, bacteria can take up extracellular DNA and integrate it into their chromosome by homologous recombination. It was conceivable that this process of transformation conferred a fitness cost. To assess whether transformation affected the competition dynamics, the Δ*rok*Δ*comEC* strain (Bs144) was generated. Deletion of *rok* increased the probability of switching into the K-state and deletion of *comEC* prevented extracellular DNA from entering the cells, inhibiting transformation (Draskovic and Dubnau, 2005). The competition dynamics of Δ*rok*Δ*comEC* against Δ*comK gfp*, were comparable to the dynamics of Δ*rok* against Δ*comK gfp* (Figure S4), indicating that transformation did not significantly affect the competition dynamics.

We further tested whether deletion of *rok* affected the competition dynamics independent of competence for transformation. To this end, we performed competition experiments between non-competent Δ*rok*Δ*comK* (Bs142) and Δ*comK gfp*. The competition showed *f*_*rok comK*_ ≈ *f*_*comK*_ for 24 h in the absence of antibiotics (Figure S5), indicating that deletion of *rok* in non-competent cells was neutral in terms of fitness under benign conditions.

In conclusion, the reduction in relative fitness associated with the K-state during outgrowth and within the stationary phase increased with increasing differentiation probability and was independent of transformation.

### Competition dynamics between strains with varying differentiation probability is strongly affected by transient exposure to antibiotics

We addressed the question how the competition dynamics were altered in the presence of antibiotics. To this end, we treated bacteria during outgrowth transiently with bactericidal and bacteriostatic antibiotics targeting different functions, namely ampicillin, erythromycin, and rifampicin.

**Figure 4:**
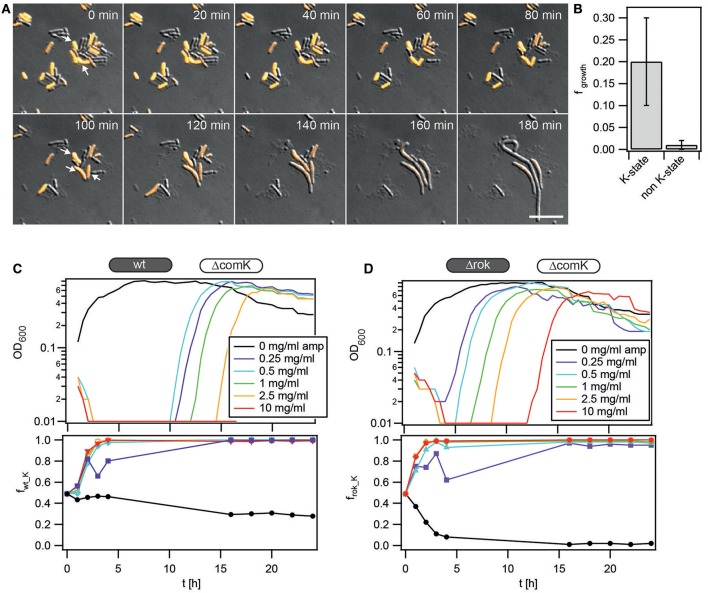
**Competence is beneficial during transient exposure to ampicillin. (A)** Typical time lapse series of *P*_*comK*_*gfp* (BD2711) outgrowth after ampicillin exposure. Bacteria were grown to T_2_, diluted and subsequently inoculated into a microscopy flow chamber. Fresh competence medium containing 0.5 mg/ml ampicillin was flushed through the flow chamber for 1 h. Afterwards, fresh competence medium without antibiotics was continuously applied, and cells were imaged. DIC and fluorescence images were merged; orange, GFP fluorescence. Arrows depict cells that eventually initiate growth. Scale bars: 10 μm. **(B)** Fractions of competent and non-competent cells that re-initiated growth (*N* = 12000 cells, error bars: standard deviation from three independent experiments). **(C,D)** Competition experiment between **(C)** wt (BD630) and Δ*comK gfp* (Bs140) and **(D)** Δ*rok* (Bs056) and Δ*comK gfp*. Competitors were grown separately to T_2_, diluted into fresh competence medium containing ampicillin as detailed in the graphs, and mixed in a 1:1 ratio, as outlined in competition assay during outgrowth. At *t* = 1 h, antibiotics were washed out and fresh competence medium was added. Upper graphs: growth curves (optical density); monitoring started at 1 h. Lower graphs: fraction of **(C)** wt and **(D)** Δ*rok* cells competing against non-competent Δ*comK gfp* cells, obtained from flow cytometry. Each graph is a representative result of at least three independent experiments.

At first, we directly visualized the dynamics of re-initiation of growth after transient exposure with antibiotics. Using the reporter strain *P*_*comK*_ *gfp*, we distinguished between K-state and non K-state cells. Cells were grown to T_2_ and subsequently inoculated between a glass cover slide and an agarose pad. The chamber was sealed by confining the agarose pad between the glass cover slide and a structured PDMS pad, as explained in the Methods. Fresh competence medium containing antibiotics was applied for 1 h. Subsequently, fresh competence medium without antibiotics was continuously supplied. Figure 4A shows a typical time lapse series succeeding treatment with ampicillin (Figure 4A, Movie S1). As expected for wt cells, a subpopulation was in the K-state at the beginning of the experiment. Non K-state cells lysed with a higher probability than the K-state cells. This observation is consistent with the time-kill kinetics in the presence of ampicillin (Figure S6). We measured the fraction of K-state and non K-state cells that eventually resumed growth (Figure 4B). On average, only 1% of initially non K-state cells resumed growth (*f*_*growth*_ = *0.01* ± *0.01*). Among the cells that were initially in the K-state, a considerable fraction re-initiated growth (*f*_*growth_K*_ = *0.2* ± *0.1*). Note that this fraction is most likely underestimated; since the GFP used as a reporter of the K-state is stable, a fraction of cells that were counted as competent may have already been in the process of escaping from the K-state when exposed to ampicillin was initiated.

Next, the competition dynamics between cells with varying probability of entering the K-state was monitored. We compared the competition dynamics of wt against Δ*comK gfp* (Figure 4C) and Δ*rok* against Δ*comK gfp* (Figure 4D). Again, the competitors were grown separately to T_2_, diluted in fresh competence medium supplemented with antibiotics, and mixed in a 1:1 ratio. After 1 h, antibiotics were washed out, and the competing strains were incubated with fresh competence medium for 24 h. During this period, the optical density of the entire population was monitored; furthermore, the fractions of competitors were monitored using flow cytometry (Figures 4C,D). With increasing concentration of ampicillin, the time period between the removal of the antibiotic and when the optical density was detectable increased (Figures 4C,D). In the experiments with the hyper-competent Δ*rok* strain (Figure 4D), these time periods were considerably shorter compared to the experiments with wt (Figure 4C). The competition dynamics of wt against Δ*comK gfp* (Figure 4C) and Δ*rok* against Δ*comK gfp* (Figure 4D) showed very similar behavior. At concentrations [amp] ≥ 0.5 mg/ml, the fractions of wt and Δ*rok* cells increased to *f*_*wt_K*_ = *1* and *f*_*rok_K*_ = *1*, indicating that the K-state conferred a selective advantage in the presence of ampicillin. At lower concentrations of ampicillin, fluctuations were observed and the final fraction was often *f*_*wt*∕*rok_K*_ < *1*. We note that quantitatively the lag phase and dynamics varied slightly from day to day and accordingly, the concentration of antibiotics at which the fraction of competent cells dominated the population (Figure S3). When comparing different strains or conditions, data from the same day were used. Moreover, the probability that the fraction of the wt competitor remained close to *f*_*wt_K*_ = *1* was variable. We attribute this observation to the fact that a small fraction of surviving Δ*comK* cells is likely to sweep through the population but is hard to detect initially.

**Figure 5:**
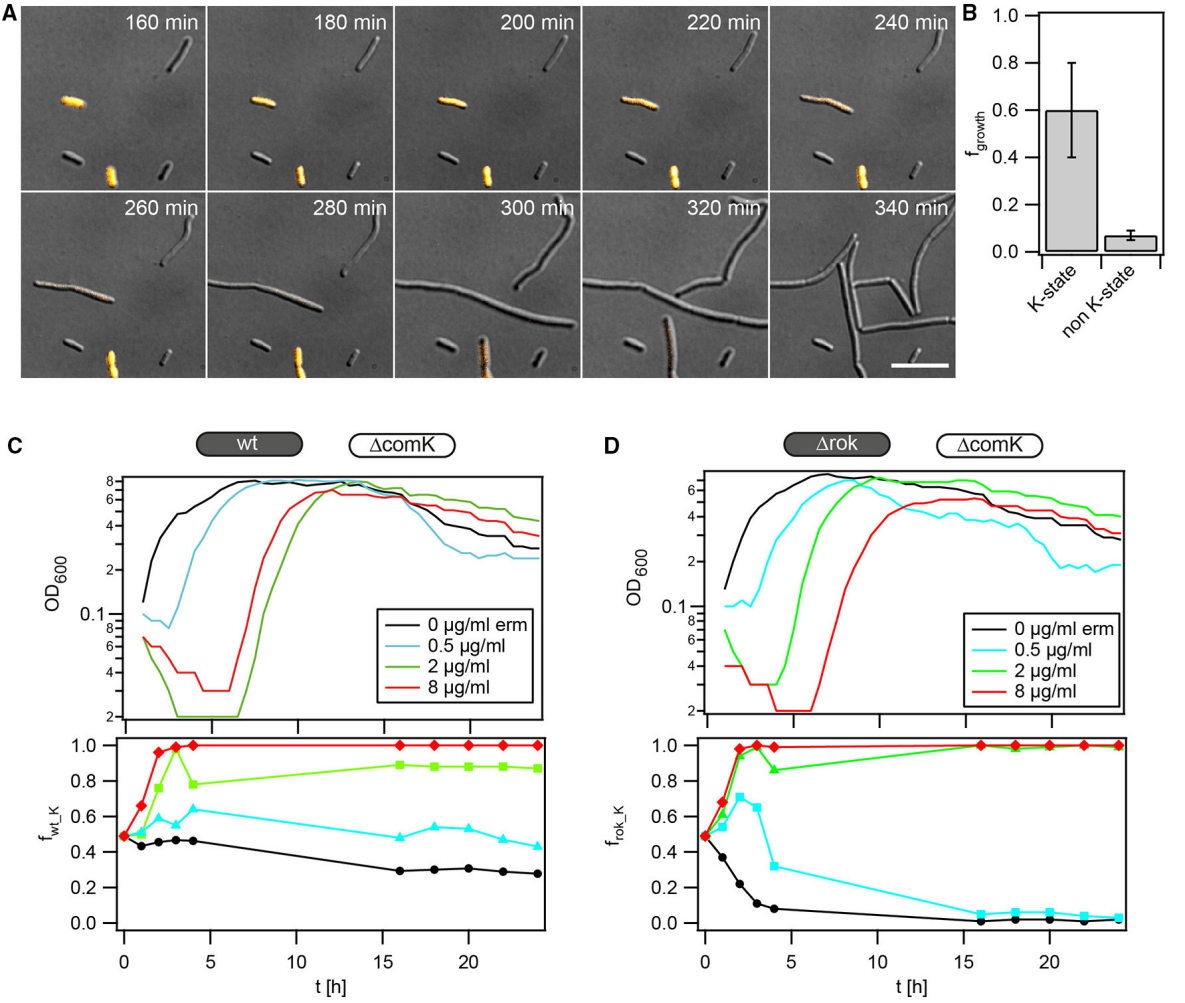
**Competence is beneficial during transient exposure to erythromycin. (A)** Typical time lapse series of *P*_*comK*_*gfp* (BD2711) outgrowth after erythromycin exposure. Bacteria were grown to T_2_, diluted and subsequently inoculated into a microscopy flow chamber. Fresh competence medium containing 8 μg/ml erythromycin was flushed through the flow chamber for 1 h. Afterwards, fresh competence medium without antibiotics was continuously applied, and cells were imaged. DIC and fluorescence images were merged; orange: GFP fluorescence. Scale bars: 10 μm **(B)** Fractions of competent and non-competent cells that re-initiated growth (*N* = 807 cells, error bars: standard deviation from three independent experiments). **(C,D)** Competition experiment between **(C)** wt (BD630) and Δ*comK gfp* (Bs140) and **(D)** Δ*rok* (Bs056) and Δ*comK gfp*. Competitors were grown separately to T_2_, diluted into fresh competence medium containing erythromycin as detailed in the graphs, and mixed in a 1:1 ratio. At *t* = 1 h, antibiotics were washed out and fresh competence medium was added. Upper graphs: growth curves (optical density). Lower graphs: fraction of **(C)** wt and **(D)** Δ*rok* cells competing against non-competent Δ*comK gfp* cells. Each graph is a representative result of at least three independent experiments.

Next, we addressed the question whether the state of competence conferred a competitive advantage during transient exposure to other antibiotics. The probability of lysis was low during and after exposure to erythromycin (Figure 5A, Movie S2). This is consistent with erythromycin being bacteriostatic. A non-negligible fraction (*f*_*growth*_ = *0.07* ± *0.02*) of non K-state cells eventually re-initiated growth (Figure 5B). However, among the cells that re-initiated growth, the fraction of initially competent cells was considerably higher (*f*_*growth_K*_ = *0.6* ± *0.2*). Subsequently, the competition dynamics of wt against Δ*comK gfp* (Figure 5C) and Δ*rok* against Δ*comK gfp* (Figure 5D) after treatment with varying concentrations of the translation-inhibitor erythromycin were compared. Both the growth curves and the competition dynamics were comparable. At [erm] = 8 μg/ml the fractions reached *f*_*wt_K*_ ≈ *1* and *f*_*rok_K*_ ≈ *1*, respectively, within 4 h. At lower concentrations of erythromycin, the fractions increased initially but decreased once the population entered the stationary phase, indicating that enough non-competent competitors were alive to take over the population.

**Figure 6:**
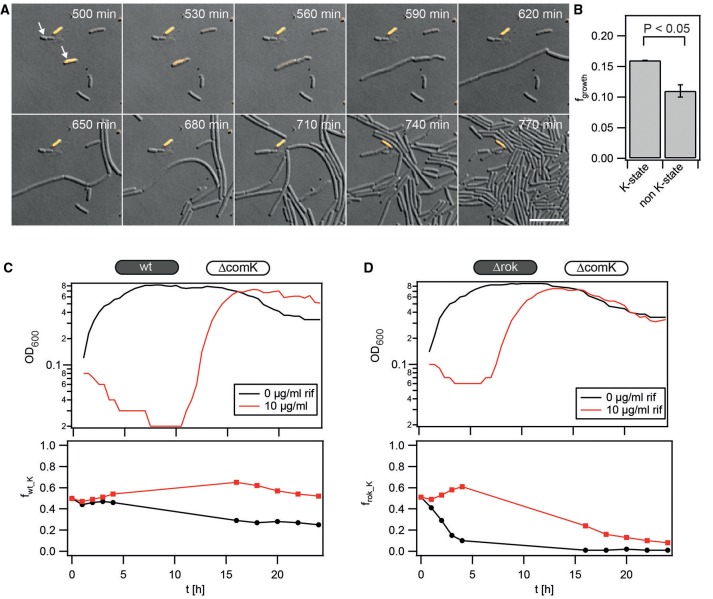
**Competence is beneficial during transient exposure to rifampicin. (A)** Typical time lapse series of *P*_*comK*_*gfp* (BD2711) outgrowth after rifampicin exposure. Bacteria were grown to T_2_, diluted and subsequently inoculated into a microscopy flow chamber. Fresh competence medium containing 10 μg/ml rifampicin was flushed through the flow chamber for 1 h. Afterwards, fresh competence medium without antibiotics was continuously applied, and cells were imaged. DIC and fluorescence images were merged; orange, GFP fluorescence. Arrows depict cells that eventually initiate growth. Scale bars: 10 μm. **(B)** Fractions of competent and non-competent cells that re-initiated growth (*N* = 3409 cells, error bars: standard deviation from three independent experiments). **(C,D)** Competition experiment between **(C)** wt (BD630) and Δ*comK gfp* (Bs140) and **(D)** Δ*rok* (Bs056) and Δ*comK gfp*. Competitors were grown separately to T_2_, diluted into fresh competence medium containing rifampicin as detailed in the graphs, and mixed in a 1:1 ratio. At *t* = 1 h, antibiotics were washed out and fresh competence medium was added. Upper graphs: growth curves (optical density). Lower graphs: fraction of **(C)** wt and **(D)** Δ*rok* cells competing against non-competent Δ*comK gfp* cells. Each graph is a representative result of at least three independent experiments.

Similarly, lysis was not prominent during or after the exposure to rifampicin (Figure 6A, Movie S3). Both competent and non-competent cells re-initiated growth with a high probability. The fraction of initially non-competent cells was *f*_*growth*_ = *0.11* ± *0.01* and the fraction of competent cells was slightly higher at *f*_*growth_K*_ = *0.161* ± *0.004* (Figure 6B). Although the difference is small it was reproducible on different days and is statistically significant according to the *t*-test applied over all cells. This small but significant benefit of competence after exposure to rifampicin is consistent with the transient increase of the fraction of wt (Figure 6C) and Δ*rok* (Figure 6D) cells during competition with Δ*comK gfp* cells.

To assess whether transformation was necessary for the selective advantage of competence in the presence of antibiotics, competition experiments between Δ*rok*Δ*comEC* and Δ*comK gfp* were performed. In the presence of ampicillin or erythromycin, Δ*rok*Δ*comEC* outcompeted the Δ*comK gfp* strain, demonstrating that tolerance against the antibiotics was independent of transformation (Figure S7). Therefore, we can exclude that DNA as a source of food was the main cause of increased fitness for the Δ*rok* bacteria. We note a slight tendency toward *f*_*rok comEC_K*_ < *f*_*rok_K*_, but the significance of this tendency is not clear.

Finally, we determined whether re-growth of the population after antibiotic exposure was caused by a beneficial mutation or by stress response. At *t* = *24* h, namely at the end of the competition experiment, cells were diluted into fresh competence medium containing the respective antibiotics at a concentration larger than their minimal inhibitory concentration (MIC). In all populations no bacterial growth was observed. This shows that the populations were sensitive to the antibiotics they were initially treated with.

We conclude that the K-state confers a benefit during transient exposure to ampicillin, erythromycin, and rifampicin. Increasing the probability of differentiation into the K-state beyond the wt level does not increase the selective advantage compared to differentiation-inhibited cells. This result indicates that the competition dynamics are very sensitive to the fraction of competent cells under benign conditions but less sensitive during antibiotic exposure.

### Stochastic differentiation is a trade-off under transient exposure to ampicillin

**Figure 7:**
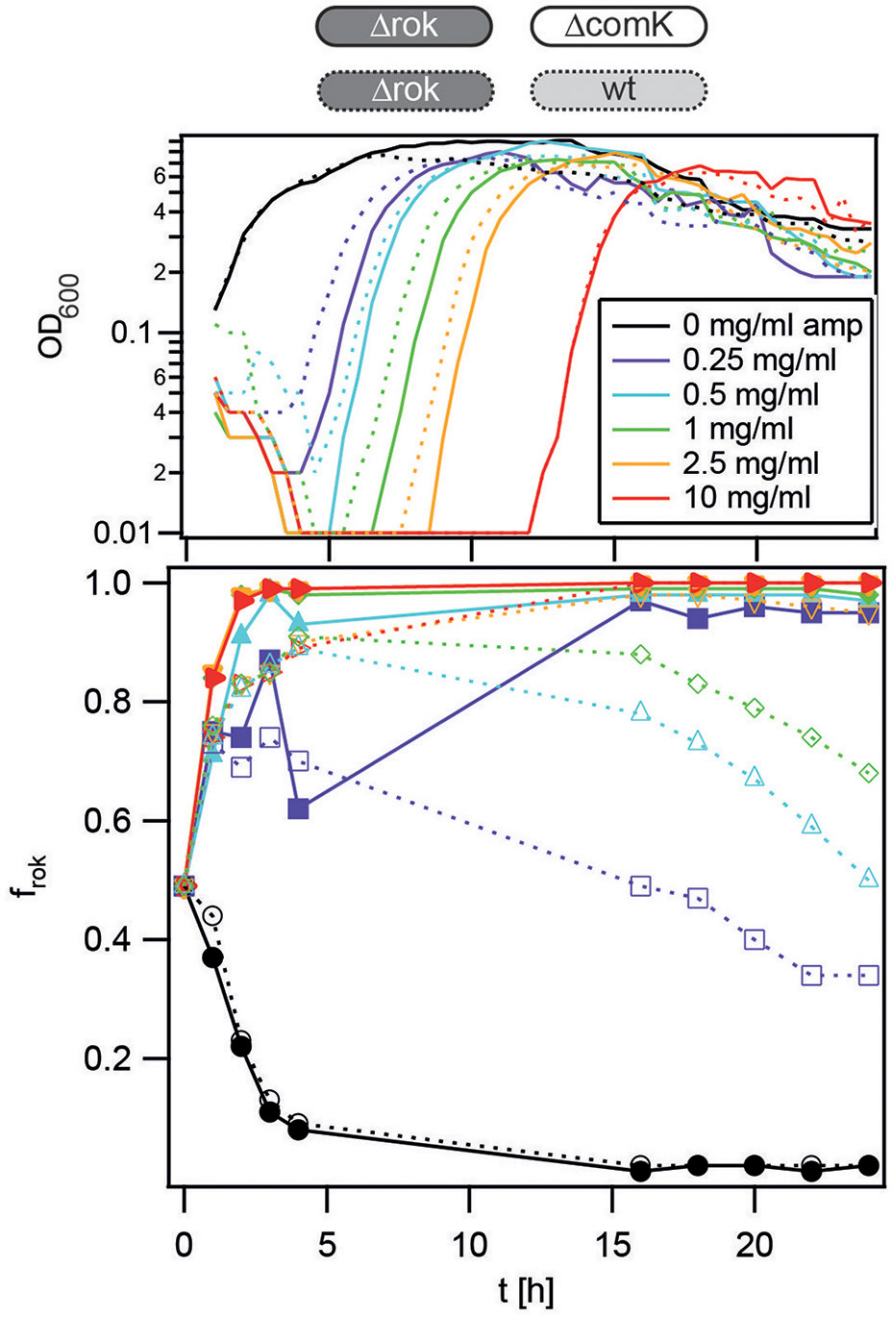
**Competition dynamics between wt and Δ***rok*** in the presence of ampicillin (amp)**. Competitors were grown separately to T_2_, diluted into fresh competence medium containing antibiotics as detailed in the graphs, and mixed in a 1:1 ratio. At *t* = 1 h, antibiotics were washed out and fresh competence medium was added. Upper graphs: growth curves (optical density of both competitors). Lower graphs: fraction of hyper-competent Δ*rok gfp* (Bs141) cells competing against Δ*comK* (Bs075, full lines, filled symbols) and Δ*rok gfp* cells competing against wt (BD630, dotted lines, open symbols). Each graph is a representative result of at least three independent experiments.

In the stationary phase, only a fraction of wt *B. subtilis* develop competence, generating phenotypic heterogeneity. This heterogeneity can be considered a fitness trade-off in fluctuating environments. Here, we have shown that the K-state confers a considerable cost in the absence of antibiotics but confers a strong benefit during transient application of antibiotics. When comparing the cost of the hyper-competent Δ*rok* strain to the wt strain (both competing with the non-competent Δ*comK gfp* strain, Figure 3) under benign conditions, the hyper-competent strain has a considerably higher cost. In the presence of antibiotics, both confer a comparable selective advantage. These experiments strongly support the idea that stochastic differentiation into the state of competence acts as a strategy for minimizing the cost of the K-state under benign conditions while generating a small fraction of competent cells as insurance under external stress. An additional experiment was performed to further support this hypothesis. Competition experiments between the hyper-competent Δ*rok gfp* strain (Bs141) and the wt strain were conducted as described above. As expected, *f*_*rok_wt*_ rose steeply directly after antibiotic exposure (Figure 7) to *f*_*rok_wt*_ = *0.9*, in agreement with a fraction of 10% competent wt cells. Within the stationary growth phase, wt cells showed a selective advantage as their fraction increased as a function of time.

This experiment shows that the hyper-competent cells have an advantage compared to the wt during antibiotic exposure but lose the competition over time, under the condition that a small fraction of wt cells survive antibiotic exposure.

### The transcription factor rok affects the competition dynamics independent of differentiation into the K-state

**Figure 8:**
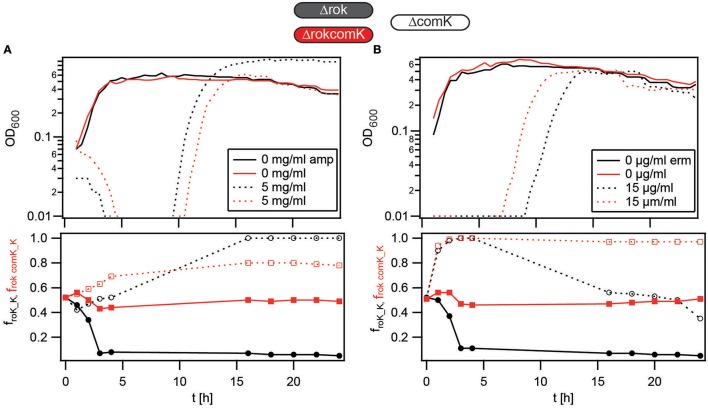
**Deletion of ***rok*** influences the competition dynamics independent of ***comK*****. Competitors were grown separately to T_2_, diluted into fresh competence medium containing antibiotics as detailed in the graphs, and mixed in a 1:1 ratio. At *t* = *1* h, antibiotics were washed out and fresh competence medium was added. **(A,B)** Upper graphs: growth curves (optical density of both competitors). Lower graphs: fraction of Δ*rok* (Bs056, black), Δ*rok comK* (Bs142, red) cells competing against non-competent Δ*comK gfp* (Bs140) cells. Each graph is a representative result of at least three independent experiments.

Rok is an important repressor of *comK* but additionally has other targets (Albano et al., 2005; Kovács and Kuipers, 2011). We tested whether deletion of *rok* affected the competition dynamics independent of differentiation into the K-state. To this end, we performed competition experiments between non-competent Δ*rok*Δ*comK* and Δ*comK gfp*. The competition showed *f*_*rok comK*_ ≈ *f*_*comK*_ for 24 h in the absence of antibiotics (Figure S5), indicating that deletion of *rok* in non-competent cells was neutral in terms of fitness under benign conditions. When ampicillin was added, the fraction *f*_*rok comK_K*_ was lower than *f*_*rok_K*_ when competing against Δ*comK gfp* under the same conditions (Figure 8A). However, *f*_*rok comK_K*_ was reproducibly > *0.5* at different concentrations of antibiotics. When erythromycin was added, we found that the *f*_*rok comK_K*_ was not decreased compared to *f*_*rok_K*_ (Figure 8B).

Since *rok* deletion affected the competition dynamics independent of the K-state, we verified that increasing ComK levels affected the competition dynamics independent of *rok*. We used the inducible *comK*_*ind*_ strain (BD3836) to increase ComK levels, where the IPTG concentration controls the fraction of K-state cells (Maamar and Dubnau, 2005). As described in the Methods section, *comK* was induced in the exponential phase, before starting the competition experiment with the wild type under transient ampicillin treatment. With transient ampicillin treatment, but without IPTG, no cell growth was observed over 3 h (Figure S8A). Still the fraction of the *comK*_*ind*_ strain rose slowly, potentially due to leaky repression of the inducible promoter. When *comK*_*ind*_ was induced, a resumption of cell growth was observed within 2 h after antibiotic treatment and the fraction of *comK*_*ind*_ cells rose to levels *f*_*ind_wt*_ ≈ *1* within 3 h, indicating that an increase of ComK levels was beneficial under transient ampicillin treatment (Figures S8B,C). The competition dynamics under transient antibiotic treatment did not depend strongly on the level of induction. Together with Figure 3D, we conclude that the ComK levels and thus the fraction of K-state cells affect the competition dynamics independent of *rok*.

In summary, when treated with antibiotics deletion of *rok* confers a selective advantage independent of the master regulator for competence, ComK.

### Sub-MIC antibiotic concentrations do not increase the probability of differentiation into the K-state

**Figure 9:**
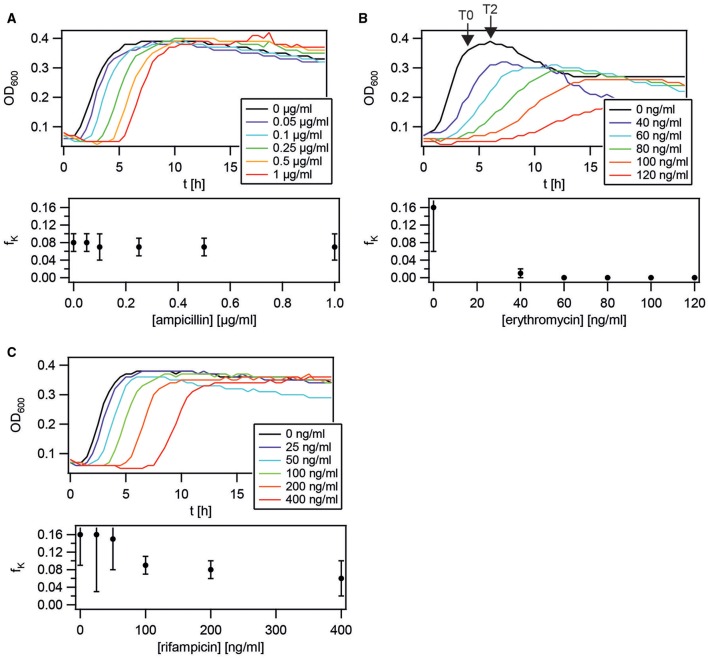
**Application of antibiotics at sub-MIC concentration does not induce competence**. wt strain with a reporter for the master regulator *comK, P*_*comK*_ *gfp* (BD2711), was diluted into fresh competence medium containing the antibiotics concentrations depicted in the graphs. Growth (OD_600_) was monitored. After 24 h the fraction of competent cells *f*_*K*_ was determined using flow cytometry. **(A)** ampicillin, **(B)** erythromycin, **(C)** rifampicin. Each graph is a representative result of at least three independent experiments.

We found that the K-state confers a strong benefit when *B. subtilis* is transiently exposed to antibiotics. Competence in *S. pneumoniae* can be induced by antibiotics at sub-MIC concentrations (Prudhomme et al., 2006; Slager et al., 2014). To assess whether antibiotics triggered competence development, *B. subtilis* expressing a *comK* reporter was grown in the presence of antibiotics. Competence development requires high cell density. To allow for cell growth and high density, experiments were performed below the minimal inhibitory concentrations of the respective antibiotics. Different antibiotics were tested on different days, accounting for the variance in the fraction of K-state cells in the absence of antibiotics. In terms of growth dynamics, we found that ampicillin and rifampicin increased the lag phase, erythromycin decreased the growth rate in the exponential phase (Figure 9). Ampicillin did not affect the fraction of competent cells *f*_*K*_. Rifampicin and gramicidin caused a slight decrease in *f*_*K*_ at elevated concentrations. In the presence of erythromycin, differentiation into the K-state was severely suppressed. We also tested novobiocin, nalidixic acid, kanamycin, gramicidin, and spectinomycin and found that none of these antibiotics increased the fraction of K-state cells.

In summary, we found no evidence of antibiotic-induced competence development.

## Discussion

### Stochastic differentiation as a fitness trade-off in fluctuating environments

Differentiation into the K-state is associated with growth-arrest (Haijema et al., 2001). Under benign conditions this growth arrest confers a fitness cost. However, in the presence of antibiotics, the K-state acts as a persister state. In its natural environment, for example in the rhizosphere, *B. subtilis* encounters various microbial species that produce antibiotics (Traxler and Kolter, 2015). Therefore, *B. subtilis* very likely benefits from the persister function of the K-state. We found that the K-state is beneficial when *B. subtilis* is transiently exposed to ampicillin, erythromycin, and rifampicin. A recent report showed that the K-state acts as a type I persister under treatment with the bactericidal drugs kanamycin and oxolinic acid (Hahn et al., 2015). Furthermore, the K-state shows persister phenotype with penicillin (Nester and Stocker, 1963). Taken together, the K-state confers a general benefit in the presence of antibiotics.

By characterizing the competition dynamics between strains with different probabilities of switching into the K-state, we quantified fitness trade-offs for differentiation into the K-state. Under benign conditions, the relative fitness of the competitors decreased with increasing differentiation probability. The frequency of the phenotypically heterogeneous wt bacteria competing with differentiation-inhibited Δ*comK* bacteria was strongly reduced after 24 h, yet its frequency amounted to 25%. However, the Δ*rok* strain (which differentiated nearly homogeneously) was reduced to a frequency of only 2%. Likewise, in competition between Δ*rok* and wt, the Δ*rok* frequency was reduced to 2% after 24 h. This comparison shows that phenotypic heterogeneity strongly reduces the decline of relative fitness caused by differentiation into the K-state. Importantly, the competition dynamics were markedly less affected by the differentiation probability in the presence of antibiotics. When comparing the time course of the fractions of wt or Δ*rok* competing with Δ*comK* cells under transient exposure to ampicillin, erythromycin, or rifampicin, they did not show a significant difference. This result indicates that a small fraction of K-state cells was sufficient for the selective advantage facing differentiation-deficient cells. When Δ*rok* bacteria competed against wt bacteria, Δ*rok* dominated immediately after antibiotic exposure because the probability of being persistent was higher. However, a considerable fraction of wt cells survived. As competition continued under benign conditions, the wt swept through the population.

Considering the pair-wise competitions under benign conditions and transient antibiotic stress, the generation of phenotypic heterogeneity by means of the K-state is a useful strategy for exploiting the persister phenotype of the K-state when under stress, while minimizing the cost under benign conditions. It is also interesting to note that this strategy of dealing with fluctuating levels of antibiotics is different for *S. pneumoniae*. *S. pneumoniae* induces competence in response to sub-MIC levels of antibiotics (Prudhomme et al., 2006; Slager et al., 2014) whereas *B. subtilis* does not, as shown in this study.

### Putative role of rok regulation in the presence of antibiotics

We chose to perform competition experiments between *rok* deletion strains and wt or *comK* deletion strains, with the aim of controlling the fraction of competent cells. The idea was that relieving repression of the master regulator of competence (*comK*) by Rok would switch most cells into the K-state, while for the wt only ~15% would switch. Although, this strategy showed the intended effect, we found that deletion of *rok* conferred an additional benefit that was independent of the K-state. In competition experiments between *rok comK* and *comK*, neither competitor was able to switch into the K-state. Nevertheless, the *rok* deletion strain showed a selective advantage when exposed transiently to ampicillin or erythromycin. Rok is an important repressor of *comK* but additionally has other targets (Albano et al., 2005; Kovács and Kuipers, 2011). These repressed genes include a number of transmembrane proteins and secreted proteins, which include antibiotics and their respective transporters. It is tempting to speculate that these proteins could increase antibiotic tolerance by transporting the antibiotics out of the cell.

## Conclusion

The K-state of *B. subtilis* confers a large cost under benign conditions, both in the stationary phase and during outgrowth; however, the K-state confers antibiotic tolerance. Because of this, the K-state is beneficial in two ways. First, it increases the probability of surviving transient antibiotic exposure. Second, this tolerance increases the window in which the bacteria can sample their environment for genetic information that confers genetic resistance by transformation. Generating phenotypic heterogeneity by means of the K-state confers a fitness trade-off minimizing the cost under benign conditions while conferring a high fitness advantage in the presence of antibiotics.

## Author contributions

All authors listed, have made substantial, direct and intellectual contribution to the work, and approved it for publication.

### Conflict of interest statement

The authors declare that the research was conducted in the absence of any commercial or financial relationships that could be construed as a potential conflict of interest.
