# Supplementary material for: Fitness Trade-Offs in Competence Differentiation of Bacillus subtilis
Source: Front Microbiol. 2016 Jun 7;7:888. doi: 10.3389/fmicb.2016.00888 (PMC4896167; doi:10.3389/fmicb.2016.00888)
Supplement: Supplementary file 4 [file DataSheet1.pdf]

Supplementary Figures for

Fitness trade-offs in competence differentiation of *Bacillus subtilis*

Melih Yüksel, Jeffrey J. Power, Jan Ribbe, Thorsten Volkmann, Berenike Maier*

Department of Physics, University of Cologne

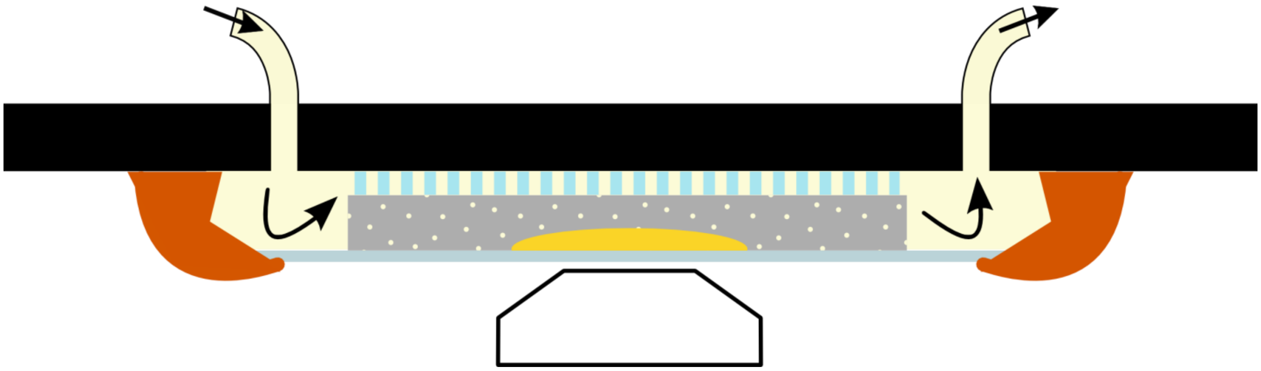

Fig. S1 Layout of the flow chamber. The sample (yellow) is sandwiched between a glass cover slide (light gray) and a porous gel (dark gray, in our setup either a PBS/agar gel or a polyacrylamide gel). The porous gel allows signaling molecules and nutrients from the medium (cream) to diffuse through to the sample but not bacteria. The porous gel rests on an array of PDMS pillars (blue) to hold the gel fixed on the cover slide. The PDMS pillars are attached to the chamber lid (black) which contains two outlets, allowing for flow injections. The cover slide is sealed to the chamber lid using picodent twinsil silicone (orange).

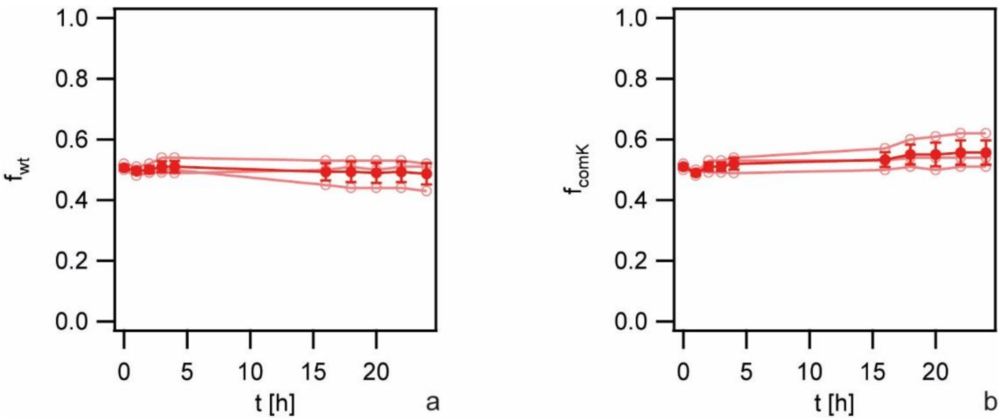

Fig. S2 *gfp* reporter does not affect the competition dynamics. Competitors were grown separately to T_2, diluted into fresh competence medium, and mixed in a 1:1 ratio. Red lines: fraction of competitor with higher probability of competence differentiation. Open circles: three independent experiments, closed circles: average and standard deviation. a) Fraction of wt (BD630) cells competed against wt *gfp* (Bs139) cells. b) Fraction of *ΔcomK* (Bs075) cells competed against *ΔcomK gfp* (Bs140) cells.

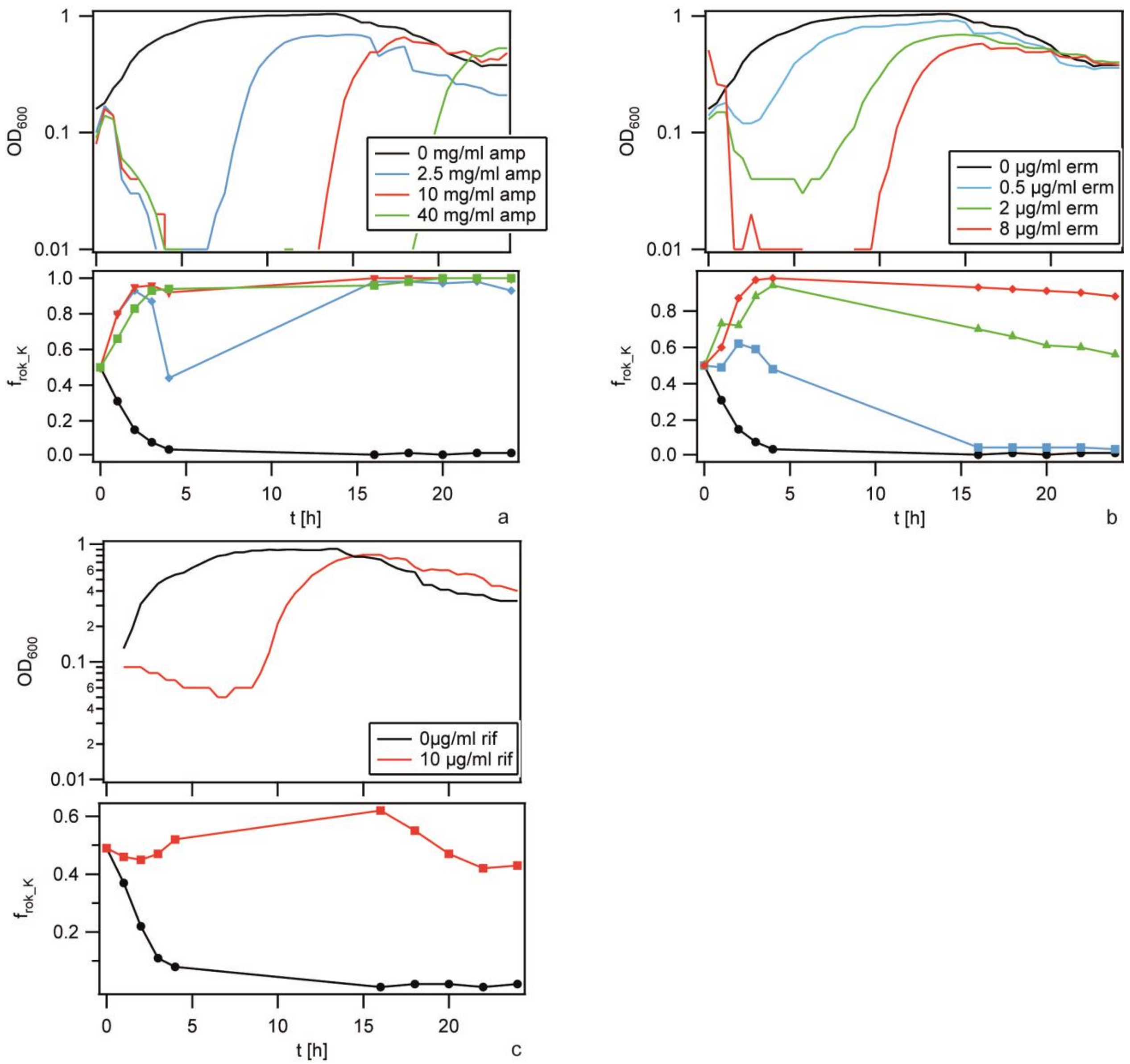

Fig. S3 Additional examples for benefit of competence during transient exposure to ampicillin and erythromycin. Competition experiment between *Δrok* (Bs056) and *ΔcomK gfp* (Bs140). Competitors were grown separately to T_2, diluted into fresh competence medium containing a) ampicillin, b) erythromycin, c) rifampicin as detailed in the graphs, and mixed in a 1:1 ratio, as outlined in competition assay during outgrowth. At $t = 1$ h, antibiotics were washed out and fresh competence medium was added. Upper graphs: growth curves (optical density); monitoring started at 1 h. Lower graphs: fraction of *Δrok* cells competing against non-competent *ΔcomK gfp* cells, obtained from flow cytometry. These graphs are additional examples (from different days) of the experiments shown in Fig. 4d, Fig. 5d, and Fig. 6d respectively.

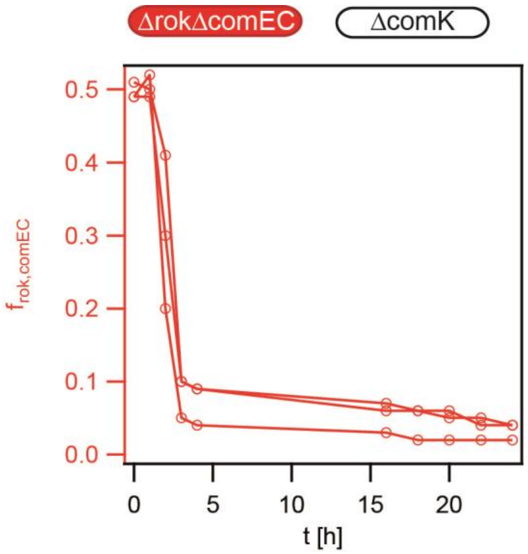

Fig. S4 The ability to transform does not affect the fitness cost of the *Δrok* strain. The competitors *ΔrokΔcomEC* (Bs144) and *ΔcomK gfp* (Bs140) were grown separately to T_2, diluted into fresh competence medium, and mixed in a 1:1 ratio. Red lines with open circles: fraction of *ΔrokΔcomEC* cells. Results of three independent experiments are shown.

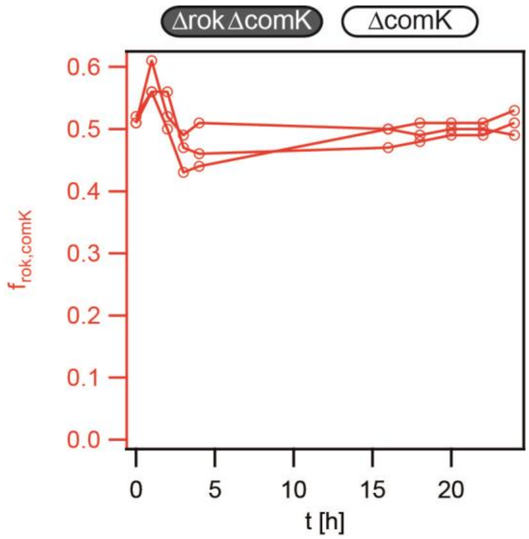

Fig. S5 The cost of *rok* deletion is related to differentiation into the K-state. The competitors *ΔrokΔcomK* (Bs142) and *ΔcomK gfp* (Bs140) were grown separately to T_2, diluted into fresh competence medium, and mixed in a 1:1 ratio. Red lines with open circles: fraction of *ΔrokΔcomK* cells. Results of three independent experiments are shown.

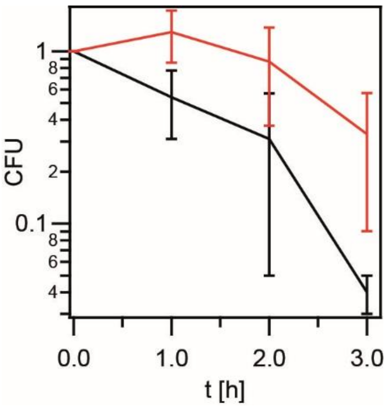

Fig. S6 Time-kill kinetics in the presence of 0.5 mg/ml ampicillin. Relative number of colony-forming units (CFU) during ampicillin treatment. Cells were diluted and plated onto agar-plates without antibiotics at different time points. The number of colonies was evaluated after 16 h. Black: *ΔcomK* (Bs075), red: *Δrok* (Bs056), error bars: standard deviation of three independent experiments.

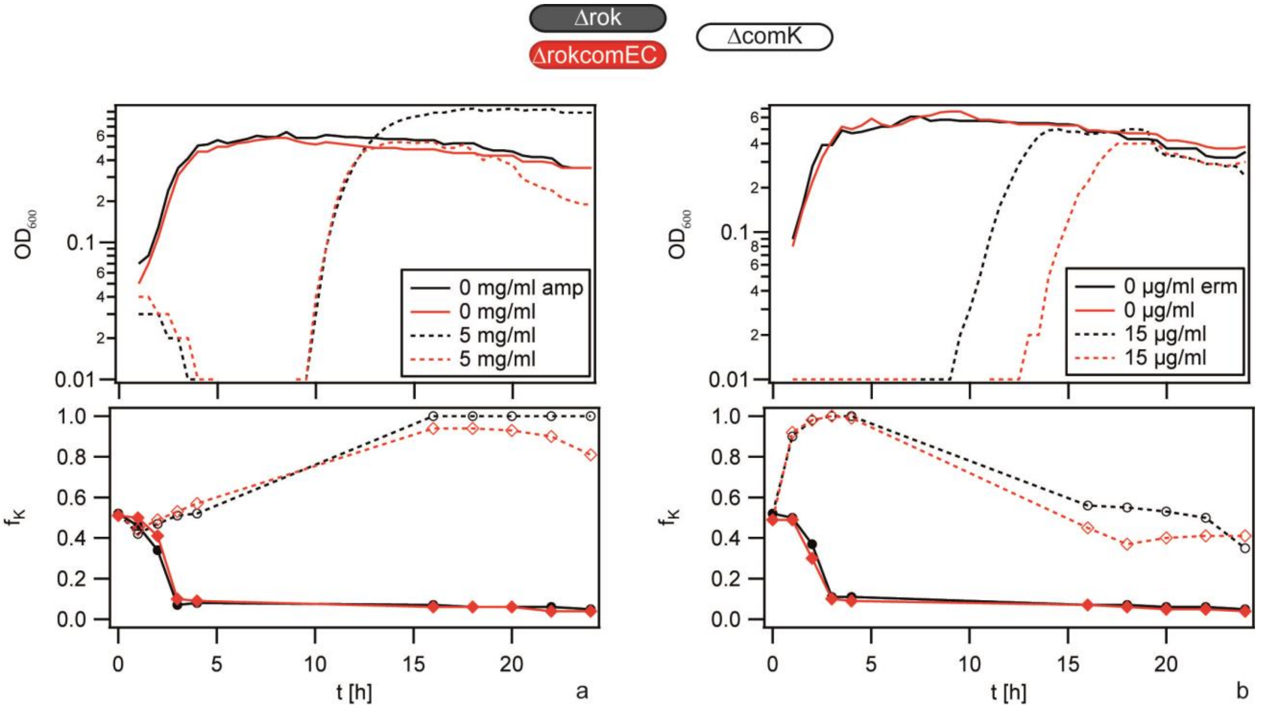

Fig. S7 The benefit of the K-state does not require transformation. Competitors were grown separately to T_2, diluted into fresh competence medium containing antibiotics as detailed in the graphs, and mixed in a 1:1 ratio. At $t = 1$ h, antibiotics were washed out and fresh competence medium was added. Upper graphs: growth curves (optical density of both competitors). Lower graphs: fraction of *Δrok* (Bs056, black), *ΔrokΔcomEC* (Bs144, red) cells competing against non-competent *ΔcomK gfp* (Bs140) cells. a) amp: ampicillin, b) erm: erythromycin. Each graph is a representative result of at least three independent experiments.

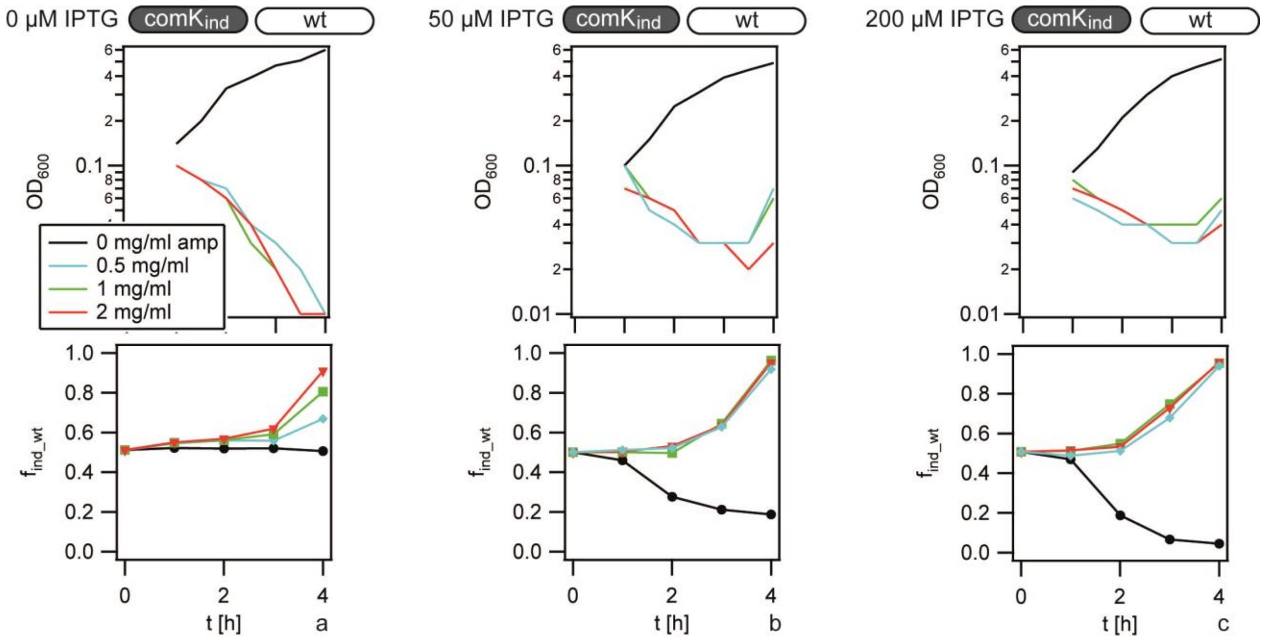

Fig. S8 Competition dynamics of wt and strain with variable *comK* induction. Competitors were grown separately for 2.5 h and induced with IPTG, as outlined in competition assay with IPTG-inducible *comK*. After IPTG was washed out, competitors were diluted into fresh competence medium containing ampicillin as detailed in the graphs, and mixed in a 1:1 ratio. At $t = 1$ h, antibiotics were washed out and fresh competence medium was added. Upper graphs: growth curves (optical density of both competitors); monitoring started at 1 h. Lower graphs: fraction of *comK*$_{\mathrm{ind}}$ (BD3836) cells competing against wt *gfp* (Bs139) cells, obtained from flow cytometry. a) No induction, b) 50 µM IPTG, c) 200 µM IPTG. Each graph is a representative result of at least three independent experiments.
